# Human T-Lymphotropic Virus-1 Associated with Adult T-Cell Lymphoma/ Leukemia and Generalized Expansion of Palatal and Jaw Bones: A Rare Case Report

**Published:** 2015-09

**Authors:** Zohreh Dalirsani, Abbas Javadzade Bolouri, Zahra Delavarian, Salma Bidad, Majid Sanatkhani, Maryam Amirchaghmaghi

**Affiliations:** 1 Dept. of Oral and Maxillofacial Medicine, Oral and Maxillofacial Diseases Research Center, Dept. of Oral Medicine, School of Dentistry, Mashhad University of Medical Sciences, Mashhad, Iran.; 2 Dept. of Oral and Maxillofacial Medicine, Dept. of Oral Medicine, School of Dentistry, Mashhad University of Medical Sciences, Mashhad, Iran.; 3 Oral Medicine Specialist, Mashhad, Iran.

**Keywords:** Human T-lymphotropic Virus-1, Leukemia-Lymphoma, Adult T-Cell, Jaw, Oral Manifestations

## Abstract

Human T-lymphotropic virus-1 (HTLV-1) can cause adult T-cell leukemia/ lymphoma (ATL/L), which is a rare and aggressive type of blood cancer.

Herein, we report a case of ATL/L in a middle-aged man with unusual jaw presentations. The patient presented with mandibular, maxillary and palatal bony hard expansion, accompanied by generalized tooth mobility six months prior to admission to the Department of Oral Medicine. The panoramic radiograph showed generalized rarefaction of jaw bones. After laboratory examinations and bone marrow aspiration, ATL/L was diagnosed in association with HTLV-1. The patient underwent chemotherapy.

Although the majority of infections associated with HTLV-1 are asymptomatic, some patients may develop blood diseases such as ATL/L and neurological disorders, mainly HTLV-1 associated myelopathy and tropical spastic paraparesis. ATL/L is a rare hematological malignancy in oral cavity that should be included in the differential diagnosis of cases with jaw swelling or generalized demineralization. Serum levels of anti-HTLV-1 antibodies should be examined in suspicious patients, particularly in endemic regions.

## Introduction

Human T-cell lymphotropic virus (HTLV-1) is a retrovirus, infecting CD4+ lymphocytes. In Iran, the main highly endemic regions for HTLV-1 are the Northeastern regions, particularly Mashhad and Neyshabur.[1] Adult T-cell leukemia/lymphoma (ATL/L) is a rare and aggressive blood malignancy, associated with HTLV-1. Therefore, the clinical manifestations and pathological characteristics of malignant lymphoma of the head and neck need to be considered in HTLV-1 endemic areas.

Proper diagnosis of ATL/L would inevitably lead to appropriate treatment and better prognosis. To the best of our knowledge, concurrent expansion of mandibular and maxillary bones has not been reported thus far; however, some cases of ATL/L accompanied by facial bone involvement have been reported.[2-3]

In this paper, we report a case of ATL/L with unusual manifestations including mandibular, maxillary, and palatal expansion as well as tooth mobility in a patient with HTLV-1.

## Case Report

The patient was a 45-year-old man who had referred to Mashhad Dental School for dental extraction. The patient had complaints of hypermobility in some teeth over the last four months. Six months earlier, he had noticed the enlargement of the jaw concurrent with pain in the muscles and bones of feet and hands after being hospitalized for appendectomy. Furthermore, the patient had a prior history of hospitalization due to fever, vomiting, nausea, and diarrhea; a diagnosis of acute urinary infection had been established.

**Figure 1:**
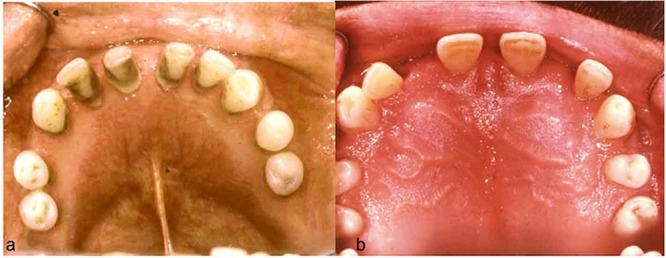
a: Mandibular bone expansion and mandibular tooth displacement  b: Palatal expansion and maxillary tooth displacement

On palpation, the swelling was non-tender, bony hard, and non-compressible with the expansion of both buccal and lingual cortical plates extending from the right second molar to the left second molar in mandibular and maxillary bones as well as hard palatal bones (Figures 1). 

The patient’s teeth were all loose and displaced. Diastema, which was observed in all teeth in both the upper and lower dental arches, appeared after jaw expansion. According to the patient's self-report, the oral mucosa was normal. We did not find any palpable lymphadenopathy in the head or neck region. 

**Figure 2:**
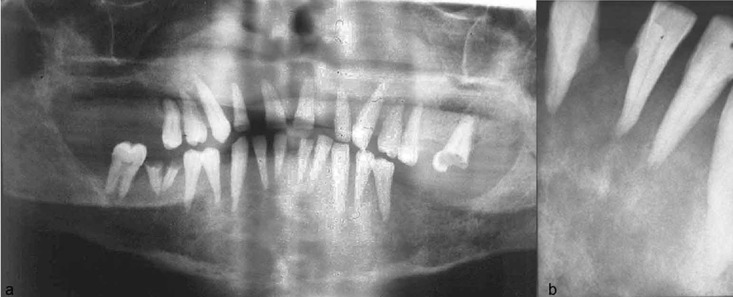
a: Panoramic radiograph showed generalized demineralization in jaw bones. The alveolar canal was unclear and the cortex was ill-defined on the right side. The posterior part of the right cortex was destroyed.  b: Lamina dura was not observed in the periapical radiograph. Severe bone loss was observed between the anterior incisors.

Radiographic and laboratory tests were performed. The panoramic radiograph revealed the generalized rarefaction of jaw bones; the inferior alveolar canal was unclear (Figure 2a). Thinning of the inferior cortex of the mandible and destruction of the posterior region of the right cortex were reported. Also, resorption of the mandibular cortex was observed on the left side.

In the midline, also the borders of maxillary sinus and hard palate were ill-defined (Figure 2b). Occlusal radiograph demonstrated the resorption of the cortical border of the anterior mandible. Periapical radiograph showed that the lamina dura was unclear and indistinct (Figure 2b).

Different laboratory tests were requested which showed a mild increase in white blood cell (WBC) count and an increase in alkaline phosphate (ALP) (Table 1). Although incisional biopsy was recommended, the patient refused to undergo biopsy.

**Table 1:** Laboratory test results in the first and second visits

**Tests**	**First visit**	**Second visit**
White blood cell	15000/μL	53900/μL
Red blood cell	5.9 M/μL	5.3 M/μL
Platelet count	392000/μL	319000/μL
Hemoglobin	15.7g/dl	12.9g/dl
Hematocrit	47%	43.6%
Alkaline phosphate	500U/L	1530U/L
Calcium	9.8mg/dl	11.4mg/dl
Phosphorus	4.4mg/dl	3.8mg/dl

After about 50 days, the patient was referred to our department again. Another blood test was ordered. As the comparison between the results of the two tests indicated, WBC count increased from 15,000/μL to 53,900/μL and ALP elevated from 500U/L to 1530 U/L. The rapid increase in ALP could be an indicator of osteolysis and increased WBC count revealed the malignant proliferation of bone marrow cells.

The differential diagnosis included different malignancies, especially hematologic disorders such as leukemia/lymphoma. The patient was referred to a hematologist and an oncologist for further evaluation. Anti-HTLV-1 antibodies were detected by means of enzyme-linked immunosorbent assay (ELISA) and Western blot analysis. 

Bone marrow aspiration showed a hypercellular bone marrow and pleomorphic lymphocytes with large flower-shaped nuclei; a high percentage of cells were atypical. A diagnosis of ATL/L was established. It seems that the first signs of this malignancy were observed in the jaw bones. The patient underwent cyclophosphamide, doxorubicin, vincristine, and prednisone (CHOP) chemotherapy.

Three years after the completion of chemotherapy, cutaneous lesions presented as maculopapular eruptions. Although the patient’s cancer was in the remission phase, he was receiving some medications. Histopathological evaluation after the incisional biopsy of cutaneous lesions showed non-specific dermatitis. According to the dermatologist’s outlook, the cutaneous lesions could be related to HTLV-1 or drug-induced dermatitis. During the patient’s visit, complete blood cell count did not show any abnormalities. 

## Discussion

HTLV-1 infection is endemic in some geographical regions and people residing in these areas may be infected by HTLV-1, without showing any clinical symptom. In these endemic regions, patients with leukemia/lymphoma should be examined for serum anti-HTLV-1 antibody level. Additionally, polymerase chain reaction (PCR) could be applied to detect the HTLV-1 genome in paraffin-embedded tissues. A previous community-based seroepidemiologic study demonstrated that the overall prevalence of HTLV-1 infection in the whole population of Mashhad city was 2.12%.[1]

Generally, most individuals infected with HTLV-1 are asymptomatic carriers; however, a few patients would present some conditions such as ATL/L or tropical spastic paraparesis which is a chronic progressive myelopathy.[4] ATL/L is a rare type of T-cell malignancy induced by HTLV-1.[5] The possibility of ATL/L development in seropositive individuals has been estimated to be less than 5% with an interval of about 30 years between acquiring the infection and development of the symptoms.[6] Nevertheless, most patients are infected by HTLV-1 earlier in their life and the risk of ATL/L development is higher in early adulthood.[7]

The mechanism involved in the malignant behavior of infected T-cells is still unknown. Disturbances in the genetic structure of host cells are proposed as risk factors for the development of oncogenic events.[8] ATL/L is characterized by systemic lymphadenopathy and/or extranodal lesions, leukemic changes in the blood, and positive retroviral serology for HTLV-1.[2, 5] Skin, liver, spleen, bone, and bone marrow are the most common sites for the involvement of extranodal ATL/L while manifestations of this cancer in the head and neck are rare.[5]

Our patient had hypercalcemia, positive serological tests for HTLV-1, and a total WBC count of 50,000/μL which are considered as indicators of ATL/L. Although other cases of ATL/L in HTLV-1-positive individuals have been reported in the literature, a few cases were initially diagnosed based on their oral manifestations.[2, 9]

In a previous study, one case of ATL/L associated with HTLV-1 was reported in which the first clinical finding was swelling of the cheek. Although dental infection was suspected, further evaluations showed that the lesion extended from the maxillary alveolus to maxillary sinus.[2] Also, a case of HTLV-1 lymphoma with maxillary sinus, nasal cavity, and bilateral orbital involvement was reported in a 48-year-old woman with normal complete blood count who responded completely to the chemotherapy treatment.[3]

In Brazil, one case of ATL/L was reported in a 30-year-old woman, presenting with a painful ulcerated lesion in the mucosa covering the hard palate. Furthermore, involvement of several bones, lungs, and axillary lymph nodes was noted.[9] However, unlike in our patient, concurrent maxillary and mandibular bone enlargement accompanied by tooth displacement was not reported in the above-mentioned cases involved with ATL/L. The current case had a normal lifestyle with no history of drug abuse and was unaware of his infection.

Furthermore, some cases of ATL/L have been reported in other parts of the body except the head and neck in non-endemic regions such as Lebanon. Two cases of ATL/L accompanied by lymphocytosis and severe hypercalcemia were reported in this region.[10] Moreover, cases of ATL/L with hepatosplenomegaly, lymphadenopathy, skin lesions, and numerous osteolytic skull lesions have been reported in a previous study.[11]

Our patient did not show any remarkable symptoms or signs exemplifying ATL/L during his first visit, similar to the mentioned cases. In our case, we considered leukemia/lymphoma in the differential diagnosis, given the generalized expansion of mandibular and maxillary bones, diffuse rarefaction of jaw, and tooth mobility. In fact, in the oral cavity, leukemia and lymphoma can cause the expansion of jaw bones, tooth mobility, and multiple ill-defined non-corticated radiolucent lesions. Additionally, non-Hodgkins lymphoma can lead to an increase in lymphocytic lactate dehydrogenase and ALP which is an indication of bone loss. 

A severe increase in ALP was found in the present case. Considering the high level of ALP, hypercalcemia, and generalized rarefaction in the patient’s jaw, hyperparathyroidism was included in the differential diagnosis. However, in hyperparathyroidism, other bones are usually involved and patients usually show other signs and symptoms of the disease.[12]

Despite the high level of ALP and generalized expansion of mandibular and maxillary bones, Paget’s disease was excluded from the diagnosis since other bones of the patient were not involved. Furthermore, if Paget’s disease only affects the maxilla or mandible, ALP remains within the normal range[13] while in our patient, an increase in ALP level was observed. Multiple myeloma was ruled out since there was not any sign or symptom such as bone pain, fever, or fatigue which are quite common in this disease.[14]

Iroi reported a case of a patient who was positive for HTLV-1 with low CD4/CD8 ratio, indicating the presence of cellular immunodeficiency.[15] Therefore, HTLV-1 infection may induce latent immunodeficiency which can increase the occurrence of lymphoma. Moreover, ATL/L with a rapid progression of leukemia and development of hypercalcemia was reported in a HIV-positive HTLV-1-positive woman.[16]

On the other hand, HTLV-1 can induce the formation of maculopapular lesions on the skin. In our patient, maculopapular lesions were observed after chemotherapy was completed. These lesions could be related to HTLV-1 complications or side-effects of chemotherapy; although, histopathologic examination of cutaneous eruption showed non-specific lesions. 

Regardless of clinical presentations, the prognosis of ATL/L is quite poor.[17] As previous studies have indicated, use of conventional chemotherapy for the treatment of ATL/L has limited benefits since HTLV-1 transformed cells are resistant to most apoptosis-inducing agents. Recent studies have shown that antiretroviral therapy, a combination of Zidovudine and interferon alpha (IFN-α), induces a high complete remission rate. Moreover, arsenic trioxide has synergistic effects with IFN-α inducing apoptosis in HTLV-1-infected T-cells.[18]

## Conclusion

ATL/L is a rare hematological disorder in the oral cavity. However, physicians and dentists should consider this malignancy in the differential diagnosis of jaw swelling or generalized rarefaction, particularly in HTLV-1 highly endemic regions. 
